# Touchscreen-based assessment of upper limb kinematics after stroke: Reliability, validity and sensitivity to motor impairment

**DOI:** 10.1186/s12984-025-01563-6

**Published:** 2025-02-11

**Authors:** Sandra Goizueta, María Dolores Navarro, Gabriela Calvo, Gloria Campos, Carolina Colomer, Enrique Noé, Roberto Llorens

**Affiliations:** 1 Neurorehabilitation and Brain Research Group, Instituto for Human-Centered Technology Research, Universitat Politècnica de València, Ciudad Politécnica de la Innovación, Building 8B, Access M, Floor 0. Camino de Vera s/n, València, 46022 Spain; 2 Instituto de Rehabilitación Neurológica, IRENEA, Fundación Vithas, València, Spain

**Keywords:** Stroke, Upper limb, Kinematic assessment, Touchscreen technology, Reliability, Convergent validity, Sensitivity

## Abstract

**Background:**

Conventional clinical tools for assessing upper limb motor function often lack the sensitivity and specificity needed to detect subtle changes in motor performance and may be subject to bias. Kinematic assessment offers a potential solution by providing objective, precise, and detailed data on movement quality. However, it is typically associated with high costs, complex equipment, time-consuming procedures, and the need for controlled environments, all of which limit its accessibility and practicality in clinical settings. This study aimed to evaluate the reliability, validity, and sensitivity of a low-cost, touchscreen-based kinematic assessment tool for measuring upper limb function in individuals post-stroke.

**Methods:**

Sixty-four individuals with stroke participated in this study. Participants performed a visually guided reaching task on a large touch screen that consisted in reaching from a central target to five outer targets arranged in a circular pattern, each at a time, and then returning to the central target. Their motor function was assessed using the *Fugl-Meyer Assessment for Upper Extremity*, the *Box and Block Test*, and the *Nine Hole Peg Test*. Kinematic measures of the trajectories performed during the reaching task were extracted and analyzed for reliability, convergent validity with clinical assessments, and sensitivity to impairment severity.

**Results:**

The kinematic measures demonstrated good to excellent test-retest reliability, with intraclass correlation coefficients ranging from moderate to excellent. The convergent validity analysis revealed multiple significant correlations between the kinematic parameters and clinical assessments, particularly in tests requiring higher skill and precision, such as the Coordination and Speed subscale of the *Fugl-Meyer Assessment for Upper Extremity* and the *Nine Hole Peg Test*. Additionally, the touchscreen-based assessment was sensitive to the severity of motor impairment, as reflected by notable differences in the kinematic measures among participants with varying levels of upper limb function.

**Conclusions:**

The touchscreen-based kinematic assessment offered an affordable yet reliable, valid, and sensitive alternative for evaluating upper limb kinematics in individuals with stroke, which could complement clinical assessments by offering additional insights into motor performance. Furthermore, its low cost, high speed, and ease of use make it a practical option for widespread clinical adoption.

**Supplementary Information:**

The online version contains supplementary material available at 10.1186/s12984-025-01563-6.

## Introduction

Approximately 70–80% of stroke survivors experience upper limb motor impairments [1], which can persist long-term, impacting their quality of life and increasing the burden on healthcare systems and caregivers [2]. Upper limb impairments post-stroke are linked to damage in the primary motor cortex and other related brain areas, which affect motor control and sensory feedback [3]. This often leads to challenges in performing coordinated movements, diminishing the ability to carry out precise tasks, and resulting in significant disability and loss of independence in daily activities [4]. Additionally, compensatory movements may develop, which can complicate rehabilitation efforts [5].

A thorough assessment of upper limb motor impairments that evaluates the ability to make rapid, selective wrist and finger movements (wrist-finger speed), efficiently manipulate small (finger dexterity) and large objects (manual dexterity), maintain arm steadiness, move the arm quickly and accurately to a target (aiming), and move the arm under continuous visual control along a path (tracking) [6, 7], is crucial for determining an accurate prognosis, designing individualized rehabilitation plans, and measuring the efficacy of those interventions. In the clinical setting, motor condition is typically assessed using observation-based scales and tests that that evaluate both task completion metrics (e.g., the number of elements grasped, moved, or placed, or the time required to perform these tasks) and motor abilities, often in an ordinal manner [8]. Clinical instruments usually offer standardized and comparable quantification of motor deficits, ensuring reliability and validity, and are relatively easy to use. However, these instruments can also have certain limitations, such as poor sensitivity to subtle changes, potential subjectivity in scoring, and significant time consumption [9]. Additionally, these instruments may lack specificity and fail to provide distinct information on the various motor components contributing to the performance [6]. Kinematic measures obtained from camera-based motion capture systems [10–12] or inertial sensors [11, 13] can potentially overcome these limitations by providing objective, precise, and detailed information about the quality of movement, such as speed, smoothness, and coordination [14, 15]. Although kinematic measures can enhance the sensitivity to detect subtle changes in motor performance that conventional scales might miss [16], they have several limitations. Kinematic analysis typically requires expensive and complex equipment and software, involves time-consuming and cumbersome setup processes, necessitates controlled environments (and often dedicated space), and uses sensors that can be uncomfortable for patients and affect their natural movement patterns. These limitations restrict the accessibility and usability of kinematic assessments in routine clinical practice [17].

Affordable equipment such as smartphones and video and depth-sensing cameras have been proposed as potential alternatives to conduct kinematic assessment at a significantly lower cost [18–21]. However, the use of multi-touch screens for this purpose remains anecdotal despite their wide availability, low cost, highly precise detection of finger touches using capacitive technology [22]. Importantly, the interaction of individuals post-stroke with relatively small multi-touch screens, such as those on tablets, has been shown to be feasible [23, 24], although dependent on the severity of their motor impairments [25]. However, only a few studies have explored the feasibility of using multi-touch devices to complement functional assessments of motor function after stroke [25–28]. Although these studies have reported varying but acceptable levels of reliability and validity, most have focused on evaluating the outcomes of upper limb movements (typically assessing whether the patient can complete the task) rather than their characteristics, with the exception of drawing exercises [26, 27]. Interestingly, a recent study compared the performance of individuals post-stroke in a visually guided reaching task using both a tablet and a robotic device, finding minimal differences between the measures obtained from each device [28]. However, the use of small screens restrict interactions to limited reaching tasks, potentially reducing the clinical relevance of the findings. Using larger screens, as employed in previous studies [29–32], could address these issues and enhance the scope and applicability of the assessments.

We hypothesized that the kinematic characteristics of a visually guided reaching task performed on a large multi-touch screen were reliable, had an acceptable convergent validity with clinical instruments, and were sensitive to impairment severity. Consequently, the objectives of this study were three-fold. First, to quantify the reliability of the kinematic assessment, as defined by the test-retest reliability, standard error of measurement, and minimal detectable change, in a representative sample of individuals with stroke. Second, to determine the convergent validity of the kinematic assessment with standardized clinical tests. Finally, to investigate the sensitivity of the app for the differentiation of post-stroke motor impairment severity.

## Methods

### Participants

All individuals with stroke who were participating in the long-term rehabilitation program at the neurorehabilitation service of Vithas Hospital El Consuelo (València, Spain) were considered potential candidates for the study. The study was conducted from January to March, 2024.

Potential candidates were included if they were over 18 years old; had a diagnosis of first ischemic or hemorrhagic stroke confirmed by computed tomography or magnetic resonance imaging; active movement in distal joints, indicated by scores above 1 on the Medical Research Council Scale for Muscle [33]; and fairly good cognitive function, defined by scores above 23 on the Mini-Mental State Examination [34]. Subjects with severe hypertonia, indicated by scores above 3 on the Modified Ashworth Scale [35]; impaired comprehension that prevented following instructions, defined by scores below 45 on the Mississippi Aphasia Screening Test [36]; severe visual or auditory impairments; and unilateral spatial neglect were excluded from the study.

Ethical approval for the study was granted by the Ethics Committee of Universitat Politècnica de València (P0924102023). All eligible candidates who agreed to take part in the study gave their written informed consent prior to their participation.

### Instrumentation

**Fig. 1:**
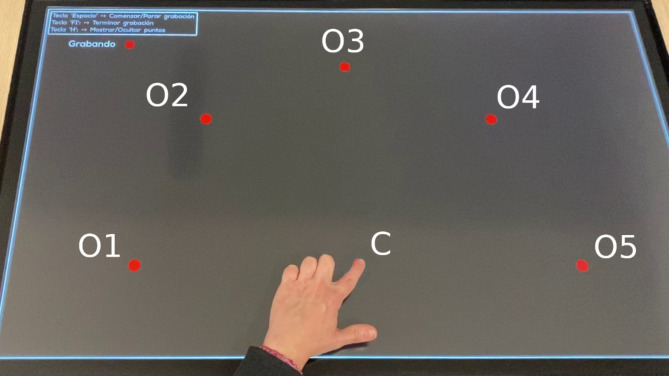
Description of the visually-guided reaching task. Participants performed a sequence of planar point-to-point reaches, beginning from a central target (C) and extending towards one of five outer targets (O1-O5) before returning to the center and moving on to the next target. The sequence followed a clockwise direction, with outer targets positioned at 0° (O1), 45° (O2), 90° (O3), 135° (O4), and 180° (O5). Participants started by reaching towards the left target (O1), returned to the center (C), then reached towards the upper-left target (O2), and continued in this manner for the remaining targets. The target points were positioned at a distance of 28 cm from the initial point

A visually guided reaching task was designed to assess aiming and tracking abilities by requiring participants to perform planar point-to-point reaches sequentially to various positions in space without pausing and while maintaining contact with the screen (Fig. 1). The task involved starting from a central target, reaching towards one of five outer targets, returning to the central target, and then moving on to the next outer target in a clockwise sequence. The outer targets were positioned at angles of 0°, 45°, 90°, 135°, and 180°, all located 28 cm from the central target. The targets were red circles with a 1 cm diameter and remained visible throughout the task.

All interactions were conducted using a 40″ touch screen, a PN-40TC1 (Sharp Corporation, Sakai, Japan), which was embedded in a standard table and positioned horizontally, parallel to the floor. The touch screen displayed the targets for the visually guided reaching task and detected finger touches at a rate of 120 Hz.

### Procedure

**Fig. 2:**
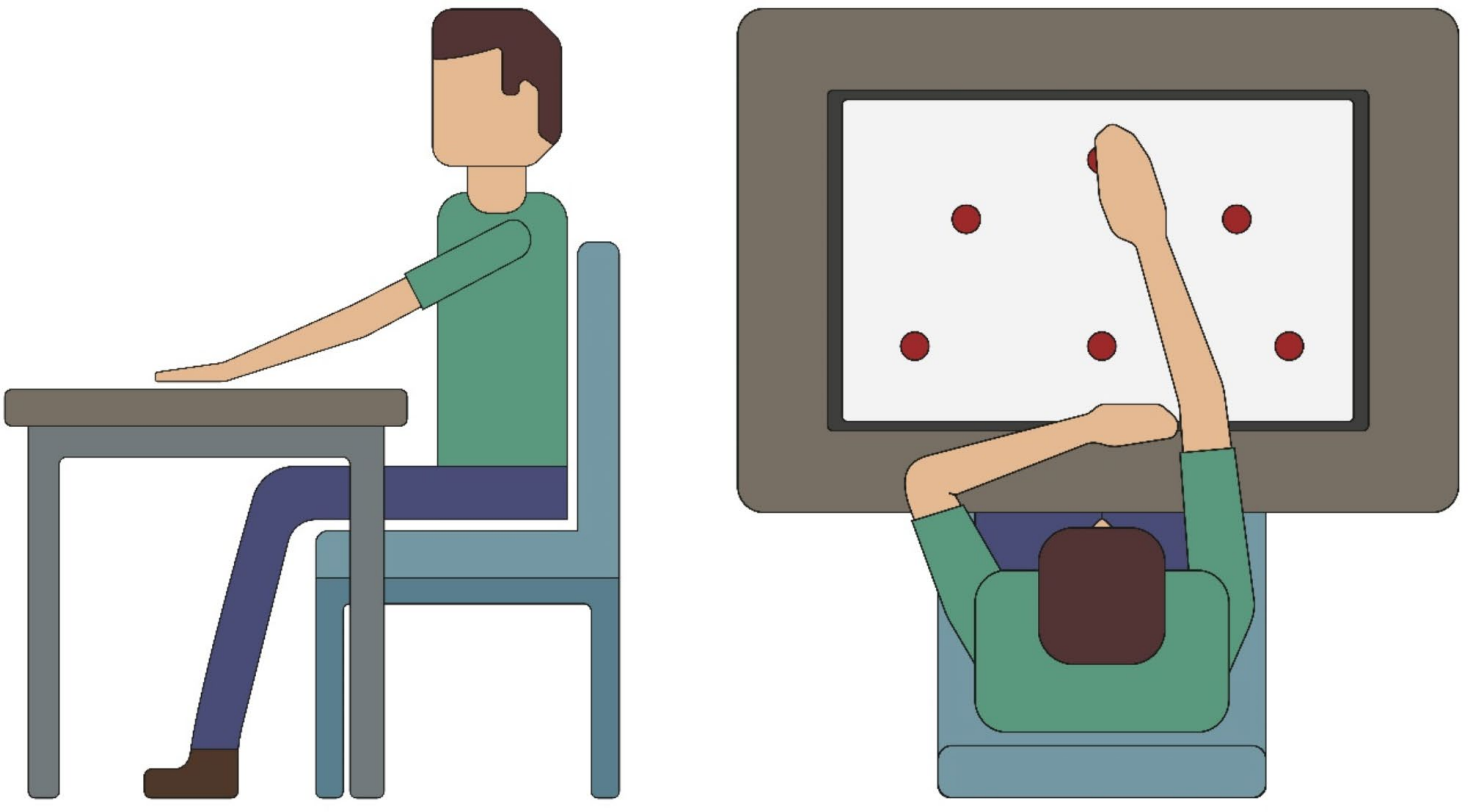
Experimenal setup. The figure shows a side view (left) and a top view (right) of a participant performing an experimental trial

The experiment took place in a dedicated room at the recruitment center, designed to be free of distractions. An experimenter conducted and supervised the sessions, guiding the participants throughout. Participants were introduced to the study and asked to sit comfortably in a chair without armrests. The chair was positioned so that their chest aligned with the central target, and the seat height was adjusted to ensure that they could comfortably reach the targets, which typically resulted in approximately 80–85 degrees of shoulder abduction (Fig. 2).

Participants were asked to complete the visually guided reaching task three times at a self-selected speed using their most impaired extremity. Before the first attempt, the experimenter demonstrated the task and provided instructions for starting each trial. The experimenter initiated and concluded each trial by pressing a button and closely supervised the participants’ performance. If any compensatory movements occurred, a tactile cue was provided to correct the participant’s performance. If this or any other issues arose during a trial, it was discarded and repeated.

In addition to the reaching task, participants’ motor function was assessed using the Fugl-Meyer Assessment for Upper Extremity [37], the Box and Block Test [38], and the Nine Hole Peg Test [39]. The Fugl-Meyer Assessment for Upper Extremity evaluates volitional movement, reflex activity, upper limb function, coordination and speed. The instrument provides a score that ranges from 0 to 66, with higher scores indicating better upper limb motor function [37]. Healthy individuals are expected to score the maximum score. The Box and Block Test assesses gross manual dexterity by measuring the number of blocks a person can transfer from one compartment of a box to another within a specified time. Typical scores for healthy adults range from 54 to 85 blocks, depending on age and hand dominance [38]. The Nine-Hole Peg Test measures fine motor dexterity by measuring the time taken to place nine pegs into holes and remove them. Lower times indicate better dexterity, with normal values for healthy individuals typically ranging between 16 and 19 s [39].

All assessments were conducted on the same day, with rest periods provided between each test.

### Data analysis

#### Signal processing

The time series data containing the *x* and *y* coordinates of the screen contacts detected by the multi-touch system were divided into five segments, each corresponding to the movement from the initial point (the center point) to each target point. Consequently, only the time records from the reaching phase were considered.

The start of the movements was defined as the first frame where the hand’s velocity exceeded 5% of its peak speed, and the end of the movements was identified as the frame closest to the target point where the hand’s velocity remained below 5% of the peak speed [40, 41].

A set of kinematic measures was selected for their ability to characterize movement performance and quality in planar movements [15, 42, 43]. These measures included parameters of movement efficiency, such as movement time and trajectory length; accuracy, including trajectory error (RMSE); speed, covering mean and maximum speed; and planning, indicated by time to maximum speed. Smoothness was assessed using the number of speed peaks (calculated from the velocity profile with a threshold of 20 mm/s), normalized mean speed, and normalized dimensionless squared jerk, computed as the mean jerk squared and normalized by the average speed [42].

#### Statistical analysis

Data normality for each parameter was assessed using the Shapiro-Wilk test. All parameters demonstrated a normal distribution except for the normalized mean speed.

Data analysis was conducted as outlined in previous studies [21, 27, 43]. Firstly, the test-retest reliability of the measures was assessed using a two-way, random-effects model intra-class correlation coefficient (ICC) with a single rater/measurement [1, 2] between the second and third trials of the visually guided reaching task. Correlations greater than 0.8 were deemed excellent, those between 0.6 and 0.8 were considered strong, and those between 0.4 and 0.6 were regarded as moderate. Correlations from 0.2 to 0.4 and below 0.2 were considered weak and very weak, respectively [44]. The first trial was discarded as it may capture initial variability due to factors like hesitation, adjusting to the movement setup, or suboptimal technique, which can skew results. Additionally, the standard error of measurement (SEM) and the minimal detectable change (MDC) were calculated [45]. The SEM quantifies the precision of individual test results, serving as a measure of reliability within individual results. The MDC, also known as the smallest detectable difference or sensitivity to change, is a statistical estimate of the smallest change detectable by a measure that signifies a noticeable change in the measurements. It represents the minimal amount of change required to exceed within-subject variability and measurement error. MDC scores higher than 30% were considered poor, from 10 to 30% were considered acceptable, and those lower than 10% were considered excellent [46]. In this study, the MDC estimates were based on a 95% confidence interval. Additionally, Bland-Altman plots were generated for each parameter to visually assess the agreement between the second and third trials. The bias, limits of agreement (LOAs), and the percentage of paired measures falling outside the LOAs were calculated. Secondly, the convergent validity of the kinematic measures obtained with the multi-touch system in comparison to clinical instruments was evaluated using Pearson or Spearman correlations. The second trial of the visually guided reaching task was considered for this purpose instead of the third trial because it could capture a familiarized, yet not fatigued, performance. It also avoided adaptation effects that might influence the third trial, where participants may further adjust their movements, potentially skewing results toward performance improvement rather than typical function. Finally, the sensitivity to motor impairment was assessed by comparing the performance of participants with the best and worst motor function in the visually guided reaching task.

All statistical analyses were conducted using Python (v3.12), with *p*-values below 0.05 considered statistically significant.

## Results

### Participants

Of the 121 individuals with stroke enrolled in the long-term rehabilitation program at the recruitment center during the recruitment period, 57 were excluded for meeting at least one of the following criteria: under 18 years of age (*n* = 5), lack of active movement in distal joints (*n* = 22), poor cognitive function (*n* = 19), severe hypertonia (*n* = 15), impaired comprehension (*n* = 14), visual or hearing impairment (*n* = 2), and visual neglect (*n* = 11). Two participants refused to participate in the study. The remaining 64 individuals post-stroke participated in the study, with no data lost from any participant. The group consisted of 25 women (39.1%) and 39 men (60.9%), with a mean age of 51.1 years (SD = 15.6) and a mean time since injury of 291.8 days (SD = 272.9). The sample included individuals with ischemic strokes (*n* = 43, 67.2%) and hemorrhagic strokes (*n* = 21, 32.8%), affecting the left hemisphere (*n* = 35, 54.7%), right hemisphere (*n* = 23, 35.9%), and brain stem (*n* = 6, 9.4%).

Participants had a mean score of 62.2 (SD = 5.2) on the *Fugl-Meyer Assessment for Upper Extremity*, 44.2 (SD = 26.8) on the *Box and Blocks Test*, and 38.5 (SD = 27.3) on the *Nine Hole Peg Test*. Additionally, their mean score on the *Functional Independence Measure* [47] was 98.2 (SD = 23.5).

All participants successfully completed three trials, each involving the task of reaching all targets and returning to the central target three times. On average, they took 17.2 s (SD = 7.0) to finish each trial, with times ranging from 6.0 s to 45.1 s.

### Reliability

#### Test-retest reliability, standard error of measurement, and minimal detectable change

All kinematic measures demonstrated good to excellent reliability (Table 1). Specifically, all measures showed excellent reliability except for trajectory length and time to maximum speed, which were strong, and the normalized mean speed, which was moderate. The highest reliability was observed for trajectory error, mean speed, and the number of speed peaks.

**Table 1:** Test-retest reliability, standard error of measurement, and minimal detectable change of the kinematic measures

Parameter		Test-retest reliability	Standard error of measurement	Minimal detectable change
*Efficiency*	*Movement time (s)*	0.86**	0.06 (4.7%)	0.16 (13.1%)
	*Trajectory length (cm)*	0.77**	0.03 (1.1%)	0.09 (3.0%)
*Accuracy*	*Trajectory error (cm)*	0.90**	< 0.01 (11.8%)	0.02 (32.7%)
*Speed*	*Mean speed (cm/s)*	0.90**	0.13 (4.3%)	0.35 (12.0%)
	*Maximum speed (cm/s)*	0.80**	0.38 (4.5%)	1.06 (12.5%)
*Planning*	*Time to maximum speed (s)*	0.66**	0.03 (6.7%)	0.09 (18.5%)
*Smoothness*	*Number of speed peaks (n)*	0.90**	2.21 (4.4%)	6.14 (12.1%)
	*Normalized mean speed*	0.55**	< 0.01 (0%)	0.02 (6.1%)
	*Normalized dimensionless squared jerk (log)*	0.81**	6.72 (2.2%)	7.17 (6.1%)

*: *p* < 0.05; **: *p* < 0.01. Moderate or stronger correlations are highlighted in bold

SEM percentages were generally low, except for trajectory error, which was also the only measure that showed poor MDC. The remaining measures had acceptable MDC values, except for trajectory length and both measures of smoothness, which were rated as excellent.

#### Agreement between measurements

**Fig. 3:**
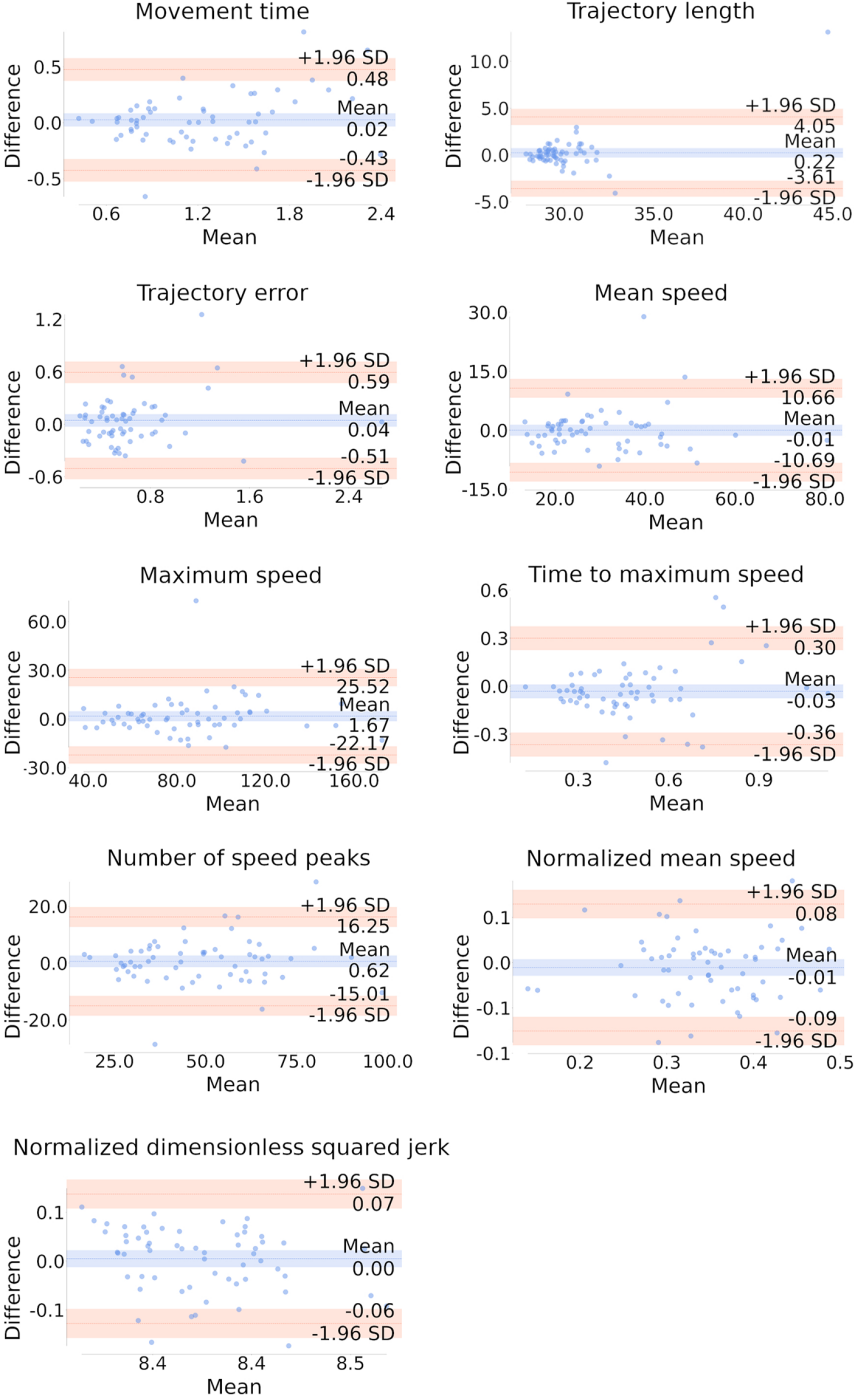
Bland-Altman plots. The Bland-Altman plots illustrate the agreement between the kinematic measures obtained in two different trials. Each plot shows the mean difference (bias) and the limits of agreement (LOA) for the following parameters: movement time, trajectory length, trajectory error, mean speed, maximum speed, time to maximum speed, number of speed peaks, normalized mean speed and normalized dimensionless squared jerk. The blue dashed lines represent the mean difference, while the pink dashed lines indicate the LOA (mean difference ± 1.96 standard deviations). The percentage of data points falling outside the LOA is also noted for each measure, indicating the level of agreement. Less than 5% of the points falling outside the LOA suggest a high level of agreement for these measures between trials

The Bland-Altman plots are shown in Fig. 3, with bias, LOAs, and the percentage of paired measures falling outside the LOAs detailed in Table 2. The number of samples falling outside the predetermined LOA was deemed acceptable for all measures, being less than 10%. Particularly, the measures for movement time, trajectory length, trajectory error, mean speed, maximum speed, and normalized dimensionless squared jerk showed especially good agreement, with fewer than 5% of paired measures falling outside the LOAs.

**Table 2:** Bias, limits of agreement, and percentage of paired measures falling outside the limits of agreement in the bland-Altman plots

Parameter		Bias	Lower LOA	Upper LOA	Measurements out of LOA
*Efficiency*	*Movement time (s)*	0.02	-0.43	0.48	4.92%
	*Trajectory length (cm)*	0.22	-3.61	4.05	3.28%
*Accuracy*	*Trajectory error (cm)*	0.04	-0.51	0.59	4.92%
*Speed*	*Mean speed (cm/s)*	-0.01	-10.69	10.66	3.28%
	*Maximum speed (cm/s)*	1.67	-22.17	25.52	1.64%
*Planning*	*Time to maximum speed (s)*	-0.03	-0.36	0.30	6.56%
*Smoothness*	*Number of speed peaks (n)*	0.62	-15.01	16.25	6.56%
	*Normalized mean speed*	-0.01	-0.09	0.08	8.20%
	*Normalized dimensionless squared jerk (log)*	0.00	-0.06	0.07	4.92%

LOA: Limit of agreement

### Convergent validity

Several significant correlations of varying strength were observed between the kinematic measures and the *Fugl-Meyer Assessment for Upper Extremity*, *Box and Block Test*, and *Nine Hole Peg Test* (Table 1). Specifically, all kinematic parameters except trajectory length demonstrated convergent validity with the *Fugl-Meyer Assessment for Upper Extremity*. Trajectory error, movement time, and the number of speed peaks showed the highest correlations, though they were only moderately strong. Movement time, mean speed, time to maximum speed, number of speed peaks, and normalized mean speed showed correlations with both the *Box and Block Test* (generally weak) and the *Nine Hole Peg Test* (moderate to strong overall). Trajectory length also showed a correlation with the *Nine Hole Peg Test*, which was notably the strongest.

**Table 3:** Convergent validity of the kinematic measures with clinical instruments

	Parameter	Fugl-Meyer Assessment for Upper Extremity					Box and block test	Nine-hole peg test
		Shoulder, elbow, forearm	Wrist	Hand	Coordination, speed	Total		
*Efficiency*	*Movement time (s)*	-0.28*	-0.50**	-0.63**	-0.55**	− 0.56**	-0.27*	0.42**
	*Trajectory length (cm)*	-	-	-	-0.45**	-	-	0.69**
*Accuracy*	*Trajectory error (cm)*	-0.53**	-0.51**	-0.50**	-0.21	− 0.60**	-	-
*Speed*	*Mean speed (cm/s)*	-	0.32**	0.36**	0.36**	0.36**	0.25*	− 0.31**
	*Maximum speed (cm/s)*	-	0.28*	0.30*	-	0.27*	-	-
*Planning*	*Time to maximum speed (s)*	-	-0.40**	-0.59**	-0.52**	− 0.45**	-0.26*	0.37**
*Smoothness*	*Number of speed peaks (n)*	-0.29*	-0.47**	-0.57**	-0.52**	− 0.54**	-0.25*	0.43**
	*Normalized mean speed*	-	-	-	0.36**	0.27*	0.66**	− 0.54**
	*Normalized dimensionless squared jerk*	-0.34**	-0.48**	-0.45**	-0.24*	− 0.48**	-	-

*: *p* < 0.05; **: *p* < 0.01. Moderate or stronger correlations are highlighted in bold

Regardless of correlation strength, the direction of the correlations consistently aligned with the constructs evaluated by the clinical instruments. This indicates that better performances on all kinematic parameters were associated with better performances on the clinical scales. For instance, longer movement times were associated with lower scores on the *Fugl-Meyer Assessment for Upper Extremity*, fewer blocks moved in the *Box and Block Test*, and more time spent completing the *Nine Hole Peg Test*. Conversely, smoother movements, as indicated by the normalized mean speed, were associated with higher scores on the *Fugl-Meyer Assessment for Upper Extremity*, more blocks moved in the *Box and Block Test*, and less time to complete the *Nine Hole Peg Test*.

### Sensitivity to motor impairment

The participant with the poorest motor function, based on her *Fugl-Meyer Assessment for Upper Extremity* score, was a 73-year-old woman who experienced an ischemic stroke in the left hemisphere 96 days prior to the study, resulting in a right hemiparesis that greatly affected her motor skills and independence. She had an *Fugl-Meyer Assessment for Upper Extremity* score of 39, moved 19 blocks in the *Box and Block Test*, and took 118 s to complete the *Nine Hole Peg Test*. Her *Functional Independence Measure* score was 68 out of 126. In contrast, the participant with the best motor function was a 51-year-old woman who also had a left ischemic stroke 314 days before the study, leading to a very mild right hemiparesis with minimal impact on her independence. She achieved the highest possible score on the *Fugl-Meyer Assessment for Upper Extremity*, moved 63 blocks in the *Box and Block Test*, and completed the *Nine Hole Peg Test* in 42 s. Her *Functional Independence Measure* score was also the maximum possible.

**Fig. 4:**
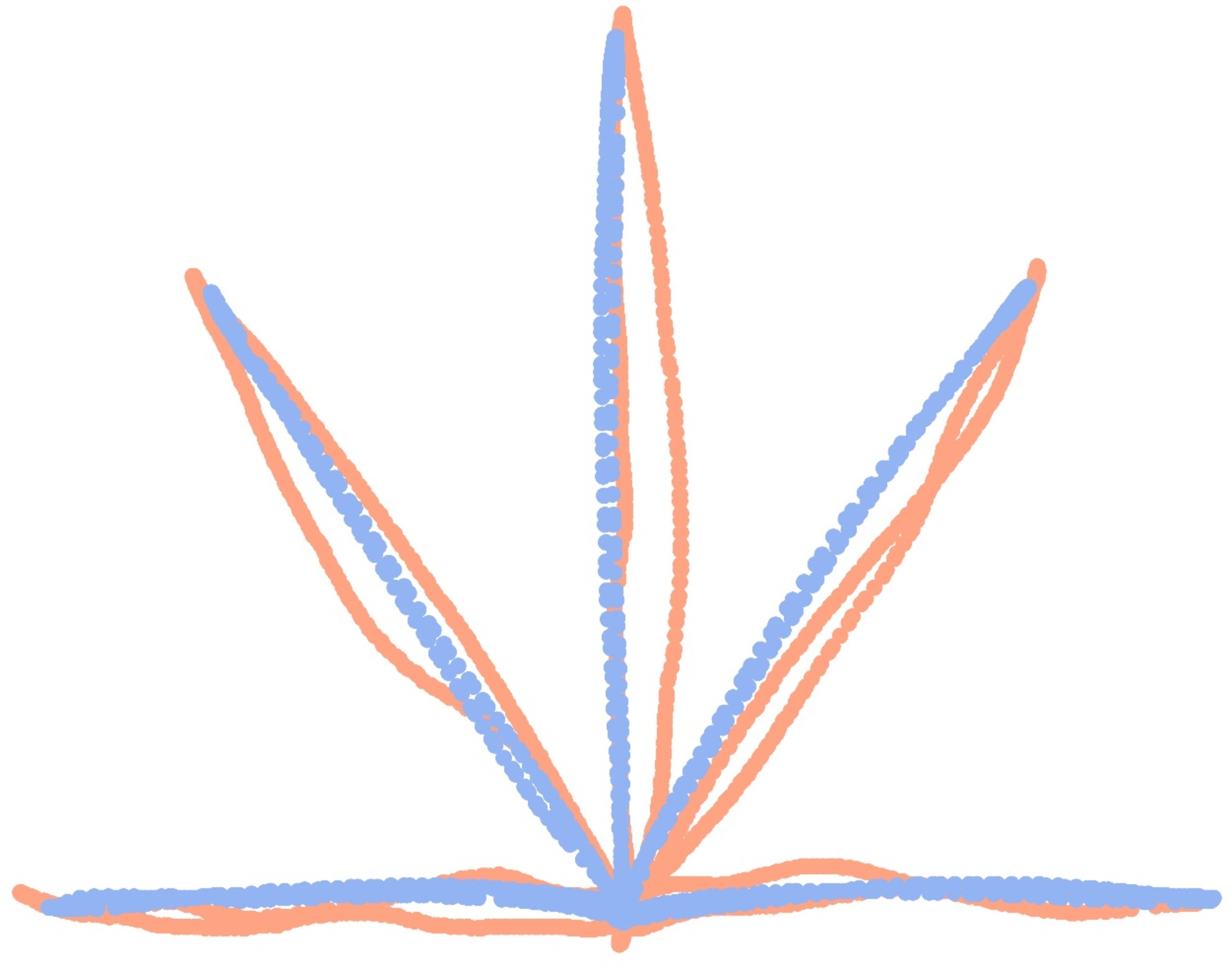
Comparison of the performance of the participants with the worst and best motor function in the visually guided reaching task. The figure illustrates the trajectories made by the participant with the worst motor function (shown in pink) and that with the best motor function (shown in blue). The trajectories of the most severely impaired participant were less linear, less smooth, and covered more distance to reach the target. In contrast, the trajectories of the less affected participant were straighter, smoother, and covered a shorter distance. The granularity of the data points also illustrates differences in movement speed between the two participants. The participant with less impairment shows trajectories with greater spacing between points, particularly in the central sections of the paths, indicating higher speed

The performance of participants with the best and worst upper limb function is illustrated in Fig. 4. The trajectories of the most severely impaired participant were more erratic, less smooth, and involved longer distances to reach the target. Conversely, the trajectories of the less affected subject were more direct, smoother, and required less distance to reach the target. The differences between participants were also evident in the kinematic parameters extracted from their movements. Consistently, worse motor function was associated with poorer values in the studied kinematic parameters. Thus, the less affected participant completed reaches in less time, with a shorter trajectory, greater accuracy, higher speed, fewer velocity peaks, and greater smoothness (Table 4).

**Table 4:** Kinematic characteristics of the participants with the best and worst upper limb function

Parameter		Participant with worst upper limb function	Participant with best upper limb function
*Efficiency*	*Movement time (s)*	1.46 ± 0.17	0.80 ± 0.13
	*Trajectory length (cm)*	29.84 ± 2.94	28.02 ± 0.67
*Accuracy*	*Trajectory error (cm)*	0.91 ± 1.08	0.55 ± 0.29
*Speed*	*Mean speed (cm/s)*	19.36 ± 2.14	33.546 ± 0.63
	*Maximum speed (cm/s)*	59.74 ± 6.48	100.91 ± 6.52
*Planning*	*Time to maximum speed (s)*	0.33 ± 0.27	0.20 ± 0.19
*Smoothness*	*Number of speed peaks (n)*	58.8 ± 0.58	30.8 ± 3.27
	*Normalized mean speed*	0.32 ± 0.04	0.36 ± 0.07
	*Normalized dimensionless squared jerk (log)*	8.78 ± 0.11	8.36 ± 0.02

## Discussion

This study evaluated the reliability, validity, and sensitivity of a touchscreen-based kinematic assessment for upper limb function in individuals post-stroke. The results demonstrated that the tool is reliable and valid, with significant correlations to conventional clinical assessments, particularly in tasks requiring precision and coordination. The tool was also sensitive to different levels of motor impairment, effectively distinguishing between participants with varying degrees of upper limb function. These findings suggest that the touchscreen-based assessment could complement existing clinical tools, providing more detailed insights into motor performance and aiding in personalized rehabilitation strategies.

The results of the test-retest reliability and the Bland-Altman analysis indicated a high level of agreement between the kinematic measures obtained in different trials, which aligns with previous research [41, 48–50]. The fact that all measurements outside the limits of agreement in the Bland-Altman analysis were under 10%, with the majority under 5%, suggests that the kinematic assessment provides consistent and reliable measurements across trials. These results highlight the consistency of the touchscreen-based assessment, particularly for key parameters of efficiency, accuracy, and speed metrics, which is crucial for both clinical practice and research, where precise and repeatable measurements are necessary for assessing progress and the effectiveness of interventions. The reliability results support the conclusion that changes in most kinematic measures reflect actual improvements or declines in motor abilities, rather than inconsistencies in measurement. Actually, given the high accuracy of multitouch technology, the objective estimation of kinematic measures, and the consistent instructions provided to participants, the reliability results are likely more reflective of variability in the participants’ performance rather than any other factors. Importantly, individuals with stroke often show higher variability when performing unilateral tasks, compared to healthy subjects, indicating a negative effect of stroke on movement consistency [51]. Kinematic measures with higher reliability, such as movement time, trajectory error, mean and maximum speed, and the number of speed peaks, appear to be more resilient to individual performance variability. In contrast, measures with lower reliability, like time to maximum speed and jerk, may be more sensitive to performance fluctuations. Therefore, it would be advisable to assess exercises with lower reliability multiple times to mitigate the impact of variability in performance. Furthermore, the lower standard error of measurement and minimal detectable change in our study, compared to 3D tasks [41, 50, 52], underscores the potential of the touchscreen-based kinematic assessment to detect true and meaningful changes in motor function, although this hypothesis requires further investigation. If confirmed in future studies, the touchscreen-based kinematic assessment could contribute to more accurate clinical and research outcomes. The differences in the complexity between planar and 3D movements likely contribute to these findings [53].

The convergent validity of the touchscreen-based kinematic assessment also align with numerous studies that have demonstrated correlations of varying strength between kinematic measures and clinical scales, particularly with the *Fugl-Meyer Assessment for Upper Extremity* [48, 49, 54–61]. The higher number and stronger correlations found with the Wrist and Hand subscales of the *Fugl-Meyer Assessment for Upper Extremity* compared to the Shoulder, Elbow, and Forearm subscale might indicate that distal functionality plays a greater role in the reaching tasks required by the kinematic assessment. However, these findings are somewhat contradictory, given that the kinematic test primarily relies on shoulder and elbow function rather than finger flexion, extension, and grasping, which are key abilities assessed by the hand subscale. This discrepancy may be explained by the differences in the recovery between distal and proximal arm function [62]. Distal motor function, such as hand and wrist movements, often shows more limited improvement compared to proximal function, like shoulder and elbow movements [63, 64]. Therefore, individuals with better distal function are likely to have overall better motor performance, which could explain the stronger convergent validity of the kinematic test with the Wrist and Hand subscales. The nature of the abilities assessed by the Coordination/Speed subscale of the *Fugl-Meyer Assessment for Upper Extremity* may account for the convergent validity of this subscale with the kinematic measures. This subscale evaluates a patient’s ability to perform rapid and smooth upper limb movements, requiring coordination across different muscle groups. Specifically, it assesses the ability to coordinate movements during tasks that demand both precision and speed. These requirements could mirror the skills measured by kinematic assessments, particularly in parameters like trajectory length and normalized mean speed, which showed correlations with the Coordination/Speed subscale but not with the other subscales. Additionally, the stronger correlations of the kinematic measures with the *Nine Hole Peg Test* compared to the *Box and Block Test* suggest that the kinematic assessment is more sensitive to fine motor control and dexterity rather than gross motor skills. This aligns with the specific demands of the *Nine Hole Peg Test*, which focuses on fine motor coordination, precision, and speed in tasks that require delicate manipulation, such as inserting and removing small pegs from holes. The kinematic measures investigated are well-suited to capturing the skills needed to excel in the *Nine Hole Peg Test*, leading to the stronger correlations observed, as previously found [57].

The significant differences in kinematic parameters between participants with the best and worst motor function, including movement time, trajectory length, and smoothness, illustrate the sensitivity of the touchscreen-based kinematic assessment to varying levels of motor impairment in stroke patients. These measures have been shown to differ between subjects with no, mild, and moderate impairments. Specifically, movement time is significantly influenced by impairment level, along with the dominance of the affected side [11]. Additionally, subjects with no impairment completed the kinematic tasks the fastest, followed by those with mild impairment, and then those with moderate impairment [11]. Significant differences in the number of speed peaks were observed between the no impairment and moderate impairment groups, as well as between the mild and moderate impairment groups [11]. Subjects with moderate stroke impairment have more pronounced deficits in kinematic measures compared to those with mild impairment. Specifically, individuals with moderate impairment had significantly longer movement times, lower peak speed, and greater difficulty in performing coordinated movements [65]. Movement time, mean speed, and peak speed effectively distinguished between groups with moderate and mild stroke impairments and healthy controls. Movement time and trajectory length also significantly increased when participants used the paretic arm to perform the task compared to the non-paretic arm [66] and have been shown to improve over time [67].

The findings of this study should be interpreted considering the following limitations. Firstly, the kinematic outcomes may depend on the specific task used. While similar visually guided reaching tasks have been frequently employed in the literature [40, 56, 68–71], different tasks might have produced different kinematic results. Secondly, the kinematic findings also depend on the specific measures analyzed in this study, and other measures could have yielded different results. However, a systematic review identified 151 kinematic metrics in stroke patients, recommending core metrics such as movement time, trajectory length, and number of velocity peaks, all of which were included in our study [15] and should facilitate the standardization and comparability of upper limb kinematic analysis in post-stroke assessments. Thirdly, using a planar movement may limit the ecological validity of the kinematic assessment. The degree to which a planar pointing task reflects real-world movement tasks is still uncertain [11], and it remains unclear if movement characteristics vary between planar and 3D tasks for the same individual. Importantly, point-to-point tasks may be particularly suitable for stroke patients with moderate to severe upper limb impairments [72]. It is also worth noting that training with a task similar to the one used in our study has shown improvements in motor function in stroke patients [40]. While our data cannot confirm the ecological validity of the task, it does demonstrate that it can be successfully performed using a touchscreen. Fourthly, in line with the above, multitouch technology only provides information on detected touch points, in this case, the hand. Therefore, it does not provide data on wrist, elbow, shoulder, or trunk joints. This information is crucial for conducting a comprehensive kinematic analysis [72], and its absence limits the analysis to the performance of the end effector. Additionally, it does not detect compensatory strategies, which could mask the functional recovery of patients [73]. Other low-cost approaches have been used to achieve full body tracking but their accuracy and reliability is limited [20]. Finally, the nature of the task and the technology used might limit its applicability to patients with moderate to mild impairments. However, it is important to note that stroke patients can interact not only with large-format touchscreens like the one used in this study [31, 32] but also with smaller screen-size tablets [26–28, 74–76].

Despite these limitations, the reliability, convergent validity, and sensitivity of the touchscreen-based kinematic assessment, combined with its ability to deliver unbiased and accurate measurements, support its potential use in examining upper limb kinematics after stroke. Its affordability and the short time required to administer the test could help overcome barriers to the widespread adoption of kinematic measures in clinical settings [17].

## Conclusion

The study demonstrated that a low-cost, touchscreen-based kinematic assessment is a reliable, valid, and sensitive tool for evaluating upper limb motor function in post-stroke individuals. The findings suggest that this method can effectively complement conventional clinical assessments, offering additional insights into motor performance. Its affordability, ease of use, and ability to capture detailed kinematic data make it a promising option for widespread clinical adoption in stroke rehabilitation settings.

## Electronic supplementary material

Below is the link to the electronic supplementary material.

Supplementary Material 1

## Data Availability

The datasets used and/or analyzed during the current study are available from the corresponding author on reasonable request.
