# Supplementary material for: Touchscreen-based assessment of upper limb kinematics after stroke: Reliability, validity and sensitivity to motor impairment
Source: J Neuroeng Rehabil. 2025 Feb 11;22:27. doi: 10.1186/s12984-025-01563-6 (PMC11817959; doi:10.1186/s12984-025-01563-6)
Supplement: Supplementary file 1 — Supplementary Material 1 [file 12984_2025_1563_MOESM1_ESM.pdf]

SPRINGER NATURE

Change of authorship request form - Journals (pre-acceptance)

Important information. Please read.

- This form should be used by authors to request any change in authorship (adding/deleting authors) including changes in corresponding authors. This form should not be used for name changes. Please fully complete all sections. Use black ink and block capitals and provide each author's full name with the given name first followed by the family name.

- By signing this declaration, all authors guarantee that the order of the authors are in accordance with their scientific contribution, if applicable as different conventions apply per discipline, and that only authors have been added who made a meaningful contribution to the work.

- Please note, in author collaborations where there is formal agreement for representing the collaboration, it is sufficient for the representative or legal guarantor (usually the corresponding author) to complete and sign the Authorship Change Form on behalf of all authors, **next to the added/removed author(s)**. **(Complete Section 3, followed by Section 6.)**
  In author collaborations where there is no formal agreement for representing the collaboration and **there are more than 10 authors**, one may sign for all, provided the signer appends correspondence that attests that each of the authors have agreed to the change **and the added/removed authors sign the form**. **(Complete Section 3, followed by Section 6.)**

- Please note, we cannot investigate or mediate any authorship disputes. If you are unable to obtain agreement from all authors (including those who you wish to be removed) you must refer the matter to your institution(s) for investigation. Please inform us if you need to do this.

- If you are not able to return a fully completed form within **30 days** of the date that it was sent to the author requesting the change, we may have to withdraw your manuscript. We cannot publish manuscripts where authorship has not been agreed by all authors (including those who have been removed).

- Incomplete forms will be rejected.

- Please return/upload this form, fully completed, to the Journals Editorial Office. The Journal and/or Publisher will consider the information you have provided to decide whether to approve the proposed change in authorship. We may decide to contact your institution for more information or undertake a further investigation, if appropriate, before making a final decision.

SPRINGER NATURE Springer Nature is one of the world's leading global research, educational and professional publishers, created in May 2015 through the combination of Nature Publishing Group, Palgrave Macmillan, Macmillan Education and Springer Science+Business Media.

SPRINGER NATURE

Change of authorship request form - Journals (pre-acceptance)

Section 1: Please provide the current title of manuscript

Manuscript ID no.: 736055ee-040f-4377-a71e-87a300311988

Title: Touchscreen-based assessment of upper limb kinematics after stroke. Reliability, validity and sensitivity to motor impairment

Section 2: Please provide the previous authorship, in the order shown on the manuscript before the changes were introduced. Please indicate the corresponding author by adding (CA) behind the name.

	First name(s)	Family name	ORCID or SCOPUS id, if available
1st author	Sandra	Goizueta	
2nd author	María Dolores	Navarro	
3rd author	Gabriela	Calvo	
4th author	Carolina	Colomer	
5th author	Enrique	Noé	
6th author	Roberto	Llorens	
7th author			
8th author			
9th author			
10th author			

Please use an additional sheet if there are more than 10 authors.

SPRINGER NATURE Springer Nature is one of the world's leading global research, educational and professional publishers, created in May 2015 through the combination of Nature Publishing Group, Palgrave Macmillan, Macmillan Education and Springer Science+Business Media.

SPRINGER NATURE

Change of authorship request form - Journals (pre-acceptance)

Section 3: Please provide a justification for change. Please use this section to explain your reasons for changing the authorship of your manuscript, e.g. what necessitated the change in authorship? Please refer to the (journal) policy pages for more information about authorship. Please explain why omitted authors were not originally included and/or why authors were removed on the submitted manuscript.

Gloria Campos has been added as an author in the revised manuscript. Due to the changes requested by the reviewers, Gloria's contributions can be considered substantial enough to meet the ICJME criteria for authorship:
https://www.icmje.org/recommendations/browse/roles-and-responsibilities/defining-the-role-of-authors-and-contributors.html
Specifically, she has contributed to data curation and visualization; and reviewing and editing the manuscript. This has been acknowledged in the Authors' Contribution subsection using the CRediT Taxonomy.

Section 4: Proposed new authorship. Please provide your new authorship list in the order you would like it to appear on the manuscript. Please indicate the corresponding author by adding (CA) behind the name. <u>If the Corresponding Author has changed, please indicate the reason under section 3</u>.

	First name(s)	Family name (this name will appear in full on the final publication and will be searchable in various abstract and indexing databases)	Affiliated institute	E-mail address
1st author	Sandra	Goizueta	Universitat Politècnica de València	sangoibe@htech.upv.es
2nd author	María Dolores	Navarro	IRENEA. Instituto de Rehabilitación Neurológica	loles.navarro@irenea.es
3rd author	Gabriela	Calvo	Universitat Politècnica de València	gcalmal@htech.upv.es
4th author	Gloria	Campos	Universitat Politècnica de València	glocamhe@htech.upv.es
5th author	Carolina	Colomer	IRENEA. Instituto de Rehabilitación Neurológica	carol.colomer@irenea.es
6th author	Enrique	Noé	IRENEA. Instituto de Rehabilitación Neurológica	enoe@comv.es
7th author	Roberto	Llorens	Universitat Politècnica de València	rllorens@htech.upv.es
8th author				
9th author				
10th author				

Please use an additional sheet if there are more than 10 authors.

SPRINGER NATURE Springer Nature is one of the world's leading global research, educational and professional publishers, created in May 2015 through the combination of Nature Publishing Group, Palgrave Macmillan, Macmillan Education and Springer Science+Business Media.

SPRINGER NATURE

Change of authorship request form - Journals (pre-acceptance)

Section 5: Author contribution, Acknowledgement and Disclosures. Please use this section to provide a new disclosure statement and, if appropriate, acknowledge any contributors who have been removed as authors and ensure you state what contribution any new authors made (if applicable per the journal or book (series) policy). Please ensure these are updated in your manuscript - after approval of the change(s) - as our production department will not transfer the information in this form to your manuscript.

New acknowledgements:

No new acknowledgements have been added

New Disclosures (financial and non-financial interests, funding):

The funding and disclosures have not changed
"This work was supported by Conselleria d'Innovació, Universitats, Ciència i Societat Digital of Generalitat Valenciana (CIDEXG/2022/15), Ministerio de Ciencia e Innovación (PID2022-141498OA-I00), and Fundació la Marató de la TV3 (60/2023)."

New Author Contributions statement (if applicable per the journal policy):

The contribution of the new author has been added as:
"Sandra Goizueta: Methodology; Software; Formal analysis; Data curation; Visualization; Writing - Review & Editing. María Dolores Navarro: Investigation; Supervision; Project administration; Writing - Review & Editing. Gabriela Calvo: Formal analysis; Writing - Review & Editing. Gloria Campos: Data curation; Visualization; Writing - Review & Editing. Carolina Colomer: Methodology; Investigation; Writing - Review & Editing. Enrique Noé: Conceptualization; Methodology; Writing - Review & Editing. Roberto Llorens: Conceptualization; Methodology; Formal analysis; Writing - Original Draft; Writing - Review & Editing; Visualization; Supervision; Funding acquisition."

State 'Not applicable' if there are no new authors.

SPRINGER NATURE Springer Nature is one of the world's leading global research, educational and professional publishers, created in May 2015 through the combination of Nature Publishing Group, Palgrave Macmillan, Macmillan Education and Springer Science+Business Media.

SPRINGER NATURE

Change of authorship request form - Journals (pre-acceptance)

Section 6: Declaration of agreement. *All* authors, unchanged, new and removed *must* sign this declaration.
(NB: Please print the form, (docu)-sign and return/upload a scanned copy. Please note that signatures that have been inserted as an image file are acceptable as long as it is handwritten. Typed names in the signature box are unacceptable.) *** Please delete as appropriate. Delete all of the bold if you were on the original authorship list and are remaining as an author.**

	First name	Family name		Signature	Date
1st author	Sandra	Goizueta	I agree to the proposed new authorship shown in section 4 **/and the addition/removal*of my name to the authorship list** /and the proposed change in corresponding author		Nov 22nd, 2024
2nd author	María Dolores	Navarro	I agree to the proposed new authorship shown in section 4 **/and the addition/removal*of my name to the authorship list** /and the proposed change in corresponding author		Nov 22nd, 2024
3rd author	Gabriela	Calvo	I agree to the proposed new authorship shown in section 4 **/and the addition/removal*of my name to the authorship list** /and the proposed change in corresponding author		Nov 22nd, 2024
4th authors	Gloria	Campos	I agree to the proposed new authorship shown in section 4 **/and the addition/removal*of my name to the authorship list** /and the proposed change in corresponding author		Nov 22nd, 2024
5th author	Carolina	Colomer	I agree to the proposed new authorship shown in section 4 **/and the addition/removal*of my name to the authorship list** /and the proposed change in corresponding author		Nov 22nd, 2024
6th author	Enrique	Noé	I agree to the proposed new authorship shown in section 4 **/and the addition/removal*of my name to the authorship list** /and the proposed change in corresponding author		Nov 22nd, 2024
7th author	Roberto	Llorens	I agree to the proposed new authorship shown in section 4 **/and the addition/removal*of my name to the authorship list** /and the proposed change in corresponding author		Nov 22nd, 2024

SPRINGER NATURE Springer Nature is one of the world's leading global research, educational and professional publishers, created in May 2015 through the combination of Nature Publishing Group, Palgrave Macmillan, Macmillan Education and Springer Science+Business Media.

SPRINGER NATURE

Change of authorship request form - Journals (pre-acceptance)

	First name	Family name		Signature	Date
8th author			I agree to the proposed new authorship shown in section 4 /**and the addition/removal*of my name to the authorship list** /and the proposed change in corresponding author		
9th author			I agree to the proposed new authorship shown in section 4 /**and the addition/removal*of my name to the authorship list** /and the proposed change in corresponding author		
10th author			I agree to the proposed new authorship shown in section 4 /**and the addition/removal*of my name to the authorship list** /and the proposed change in corresponding author		

Please use an additional sheet if there are more than 10 authors.

In case of author collaborations with formal agreement:

	Name of consortium/consortia	First name	Family name		Signature	Date
Representative/legal guarantor				I agree to the proposed new authorship shown in section 4 /**and the addition/removal*of my name to the authorship list** /and the proposed change in corresponding author		

Both added/removed authors should complete the information in the first table under Section 6.

---- End of form ----

SPRINGER NATURE Springer Nature is one of the world's leading global research, educational and professional publishers, created in May 2015 through the combination of Nature Publishing Group, Palgrave Macmillan, Macmillan Education and Springer Science+Business Media.
